# GenoTypeMapper: graphical genotyping on genetic and sequence-based maps

**DOI:** 10.1186/s13007-020-00665-7

**Published:** 2020-09-10

**Authors:** Mathieu Deblieck, Andrii Fatiukha, Norbert Grundman, Lianne Merchuk-Ovnat, Yehoshua Saranga, Tamar Krugman, Klaus Pillen, Albrecht Serfling, Wojciech Makalowski, Frank Ordon, Dragan Perovic

**Affiliations:** 1 Institute for Resistance Research and Stress Tolerance, Julius Kühn-Institute, Erwin-Baur-Str. 27, 06484 Quedlinburg, Germany; 2 Institute of Evolution and Department of Environmental and Evolutionary Biology, University of Haifa, Abba Khoushy Ave 199, 3498838 Haifa, Israel; 3 Faculty of Medicine, Institute of Bioinformatics, Westfälische Wilhelms-Universität Münster, Niels-Stensen Strasse 14, 48149 Münster, Germany; 4 Robert H. Smith Faculty of Agriculture, Food and Environment, The Hebrew University of Jerusalem, POB 12, 76100 Rehovot, Israel; 5 Institute of Agricultural and Nutritional Sciences, Department of Plant Breeding, Martin Luther University Halle-Wittenberg, Betty-Heimann-Str. 3, 06120 Halle, Germany

**Keywords:** Genetic maps, Sequence-based maps, Graphical genotyping, Molecular breeding, Algorithm

## Abstract

**Background:**

The rising availability of assemblies of large genomes (e.g. bread and durum wheat, barley) and their annotations deliver the basis to graphically present genome organization of parents and progenies on a physical scale. Genetic maps are a very important tool for breeders but often represent distorted models of the actual chromosomes, e.g., in centromeric and telomeric regions. This biased picture might lead to imprecise assumptions and estimations about the size and complexity of genetic regions and the selection of suitable molecular markers for the incorporation of traits in breeding populations or near-isogenic lines (NILs). Some software packages allow the graphical illustration of genotypic data, but to the best of our knowledge, suitable software packages that allow the comparison of genotypic data on the physical and genetic scale are currently unavailable.

**Results:**

We developed a simple Java-based-software called GenoTypeMapper (GTM) for comparing genotypic data on genetic and physical maps and tested it for effectiveness on data of two NILs that carry QTL-regions for drought stress tolerance from wild emmer on chromosome 2BS and 7AS. Both NILs were more tolerant to drought stress than their recurrent parents but exhibited additional undesirable traits such as delayed heading time.

**Conclusions:**

In this article, we illustrate that the software easily allows users to display and identify additional chromosomal introgressions in both NILs originating from the wild emmer parent. The ability to detect and diminish linkage drag can be of particular interest for pre-breeding purposes and the developed software is a well-suited tool in this respect. The software is based on a simple allele-matching algorithm between the offspring and parents of a crossing scheme. Despite this simple approach, GTM seems to be the only software that allows us to analyse, illustrate and compare genotypic data of offspring of different crossing schemes with up to four parents in two different maps. So far, up to 500 individuals with a maximum number of 50,000 markers can be examined with the software. The main limitation that hampers the performance of the software is the number of markers that are examined in parallel. Since each individual must be analysed separately, a maximum of ten individuals can currently be displayed in a single run. On a computer with an Intel five processor of the 8th generation, GTM can reliably either analyse a single individual with up to 12,000 markers or ten individuals with up to 3,600 markers in less than five seconds. Future work aims to improve the performance of the software so that more complex crossing schemes with more parents and more markers can be analysed.

## Background

High-throughput plant genotyping technologies enable the testing of a few thousands to hundreds of thousands of markers in large numbers of samples in parallel. Such technologies include next-generation sequencing (NGS) and array-based technologies, such as the Diversity Array Technology (DArT) [1], genotyping by sequencing (GBS) [2], the Illumina Bead Array/ Infinium Technology [3, 4] or the Affymetrix Gene Chip/Axiom Technology [5, 6]. Predesigned Illumina Bead Arrays and Affymetrix chips do exist for numerous important organisms and crops, such as apples, barley, brassica, cherry, chickpea, cotton, cowpea, grape, lettuce, maize, oat, peach, pear, peanut, pepper, potato, rice, rose, rye, ryegrass, soybean, strawberry, sunflower, tomato and wheat [7].

Notably, only a fraction of this information can be included in a genetic map due to several limitations. The resolution of a genetic map depends on the number of progenies analysed, the number of recombination events observed between polymorphic markers [8, 9] and the number of markers along the chromosomes [10]. Markers that do not show recombination events are assigned to the same genetic position; hence, the distances of genetic and physical maps may differ significantly from each other, especially close to centromeric and telomeric regions [11–13]. Genetic maps often serve as templates for the localization of major genes and quantitative traits loci (QTLs) on chromosomes. The identification of such regions depends on differences in phenotypic data that can be assigned to recombination events observed between different markers [14]. Probability of meiosis and recombination events correlate with the number of individuals in a population. Therefore, the number of markers that are used to construct a genetic map can be adjusted to the size of the mapping population and the number of expected recombination events [14]. The biased picture of a genetic map might lead to imprecise assumptions and estimations about the size and complexity of genetic regions and the selection of suitable molecular markers for the incorporation of traits in breeding populations or near-isogenic lines (NILs). Furthermore, genotypic data that cannot be used for the construction of a genetic map, e.g., monomorphic, heterozygous or failed data in one of the parents of a biparental mapping population, are traditionally discarded at a very early stage of the mapping procedure.

The recently published physical pseudomolecules of cereals, such as einkorn (*Triticum monoccum)* (4.94 Gb.) [15], wild emmer wheat (*T. turgidum* ssp. *dicoccoides*) (12 Gb) [16], durum wheat (*T. turgidum* ssp. *durum*) [17] goat grass (*Aegilops tauschii*) (4.3 Gb.) [18], barley (*Hordeum vulgare*) (4.75 Gb.) [19] and bread wheat (*T. aestivum*) (15.4–15.8 Gb) [20, 21] deliver a valuable tool to solve this distorted picture and to include missing genotypic information to finally gain a better understanding of the physical scale of the regions of interest. Software packages such as Graphical Genotypes (GGT) [22] and Flapjack [23] or complex genome browsers such as the Integrative genome viewer (IGV) [24] allow the graphical illustration of genotypic data, but do not allow the comparison of genotypic data of offspring of different crossing schemes in different maps. The same is true for software packages that focus on the detection and illustration of specific genomic introgressions on the genetic or physical scale, like IView [25] or the Physical Introgression Browser [26]. Tools such as the R-based MareyMap [27] allow the comparison of genetic and physical maps but do not allow analysis of genotypic data. These consequently cannot consider the distortions between physical and genetic maps and therefore miss the information that can be obtained by integrating additional genotypic information from equivalent physical regions into the genetic map, i.e., heterozygous markers. We therefore developed a combined approach that allows graphical genotyping on the physical and genetic scale and implemented it in a small platform-independent Java application, named GenoTypeMapper (GTM). The software was developed and tested under Windows 10 but should run on any operating system (OS) that has the Java virtual machine (JVM) installed.

GTM allows users to filter different allelic types (e.g., heterozygous alleles) to plot genotypic data in different ways to obtain a customized view of genomic and/ or genetic regions and their allelic composition.

Here, we present the software in detail followed by results obtained on SNP genotyping information of two NILs (NIL-U-2B-1, NIL-B-7A-2) that carry QTL-regions for improved drought stress tolerance on chromosome 2BS and 7AS from the drought tolerant wild emmer accession # G18-16 [28]. Both NILs were shown to be more tolerant to drought stress than their recurrent parents but show phenotypes that may stem from linkage drag, e.g., an increased plant height or an increased number of days to heading (DPH) [28, 29]. We used GTM to perform genotypic analysis on the genetic and physical scales of both NILs and the respective recombinant inbred lines (RILs) (see Additional file 1: Figure S1) [30–32].

## Implementation

GTM was developed and tested under Windows 10 but should run on any OS that has the JVM installed. A simple JAR file of the program, a setup file for windows, test data (template files) and a small quick-start tutorial are provided on www.genotypemapper.org. The test data includes the original genotypic data from the F2 plants, NILs and F2 pig individuals that are discussed in the article. Additionally, extra test data sets are provided on the website to test further options for GTM, e.g., the comparison of two genetic maps. More details about the data are also summarized in a small ReadMe file on the website.

### Input data

Input data can be loaded in the form of a GTM-specific tab delimited text file with at least nine columns (Table 1).

**Table 1:** Standard input data for GTM

Chr	Marker	*Genetic position*	*Physical or genetic position*	*Lod-value*	*P1*	*P2*	*P3*	*P4*
**1A**	**SNP_1**	*0.00*	*12,000*	*3.0*	*A*	*T*	*A*	*A*

The input data format always requires nine columns. Mandatory information is written in bold, additional information in italics. Information that is not available should be indicated with “NA”. Please note that the chromosome, the marker name, and a physical and/or genetic position must be provided to the program. For more detailed information, see the text.

The first two columns of the GTM input file contain chromosome and marker names, and the third and fourth column contain physical and/or genetic positions of the markers.

**Fig. 1:**
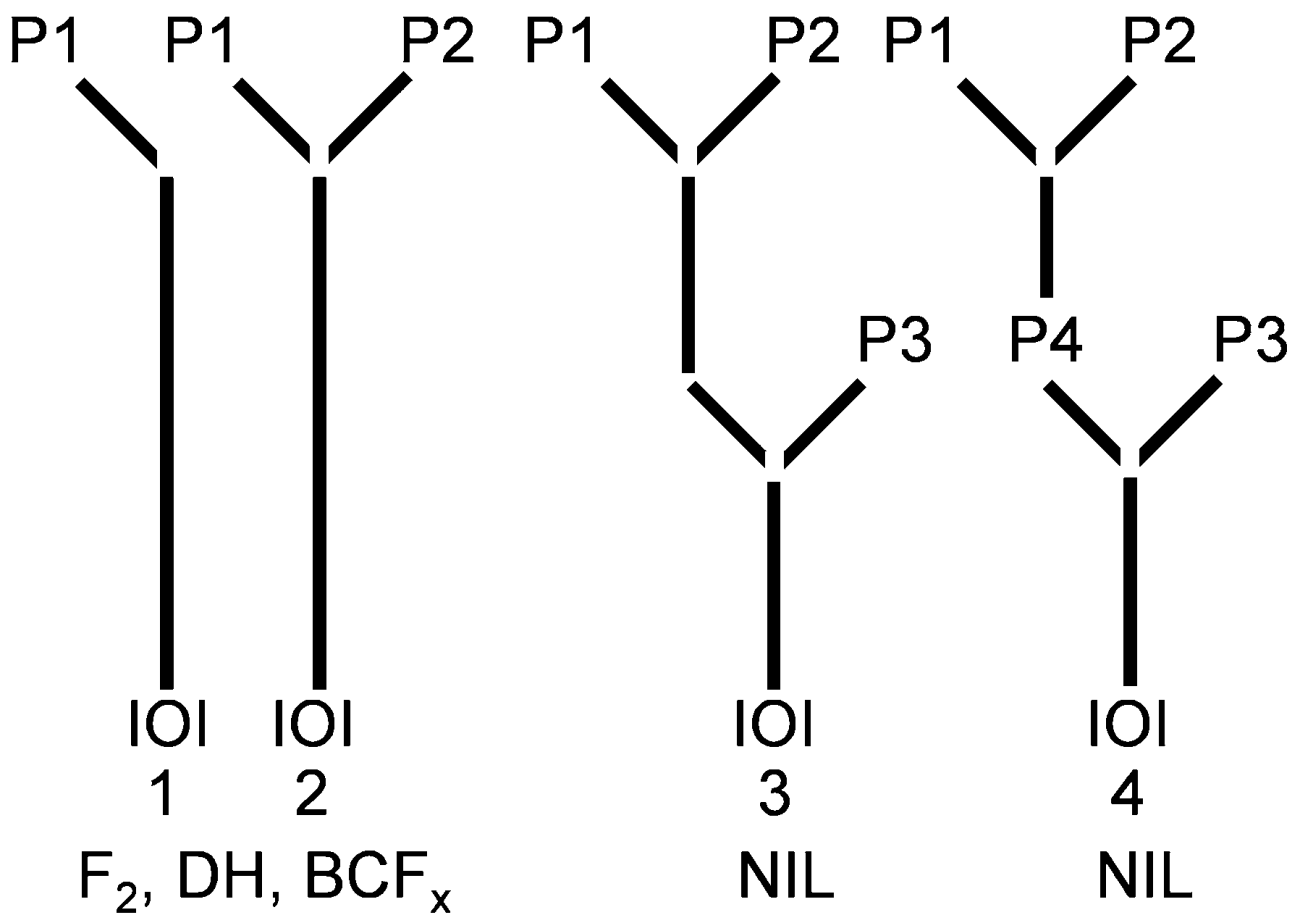
Crossing schemes which can be implemented in the background of the mapping software. Parental lines of the genotype analyzed (IOI) are abbreviated by „P “, numbers 1–4 show the possible number of parental lines. Eligible scope of applications can be double haploids (DH), F2-populations (F2), Back cross populations (BCFx) and near isogenic lines (NIL)

Column five can contain logarithm of odds (LOD) information, and columns six and seven are dedicated to genotype information from the parents of a typical bi-parental population, e.g., a Double Haploid (DH) or F2 population (Fig. 1). Recurrent parents and individuals, which are used for the establishment of NILs (Fig. 1), can be specified in the eighth and ninth columns. Genotypic data of the remaining accessions can be added to the tenth and following columns. Please note that example input files are available on www.genotypemapper.org.

### User interface (UI)

**Fig. 2:**
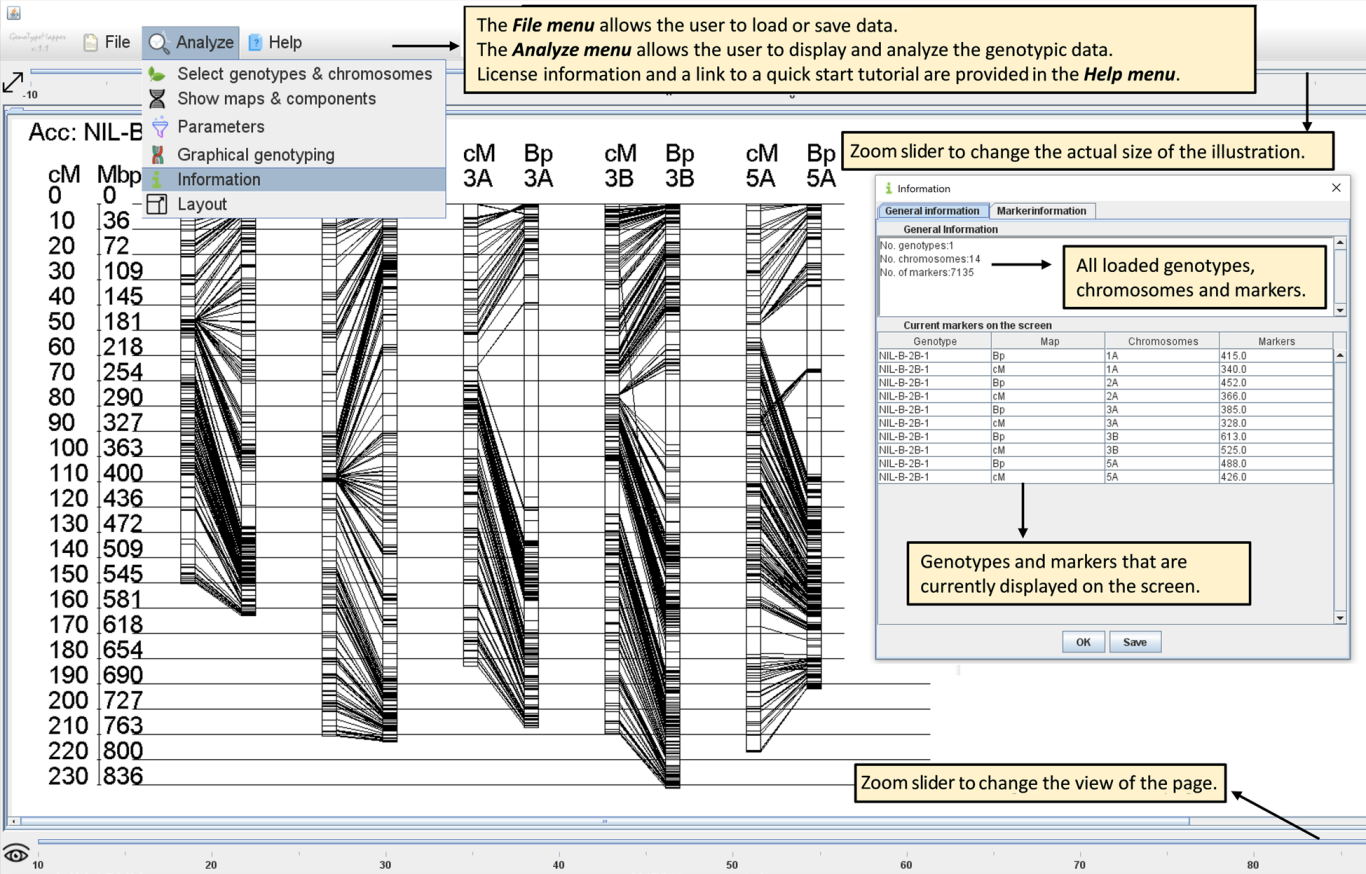
Graphical user interface of GenoTypeMapper in default mode. In the default mode, multiple chromosomes of up to ten individuals can be displayed. The menu item “information” allows the display of the number of chromosomes, genotypes and markers, that were uploaded to the software

**Fig. 3:**
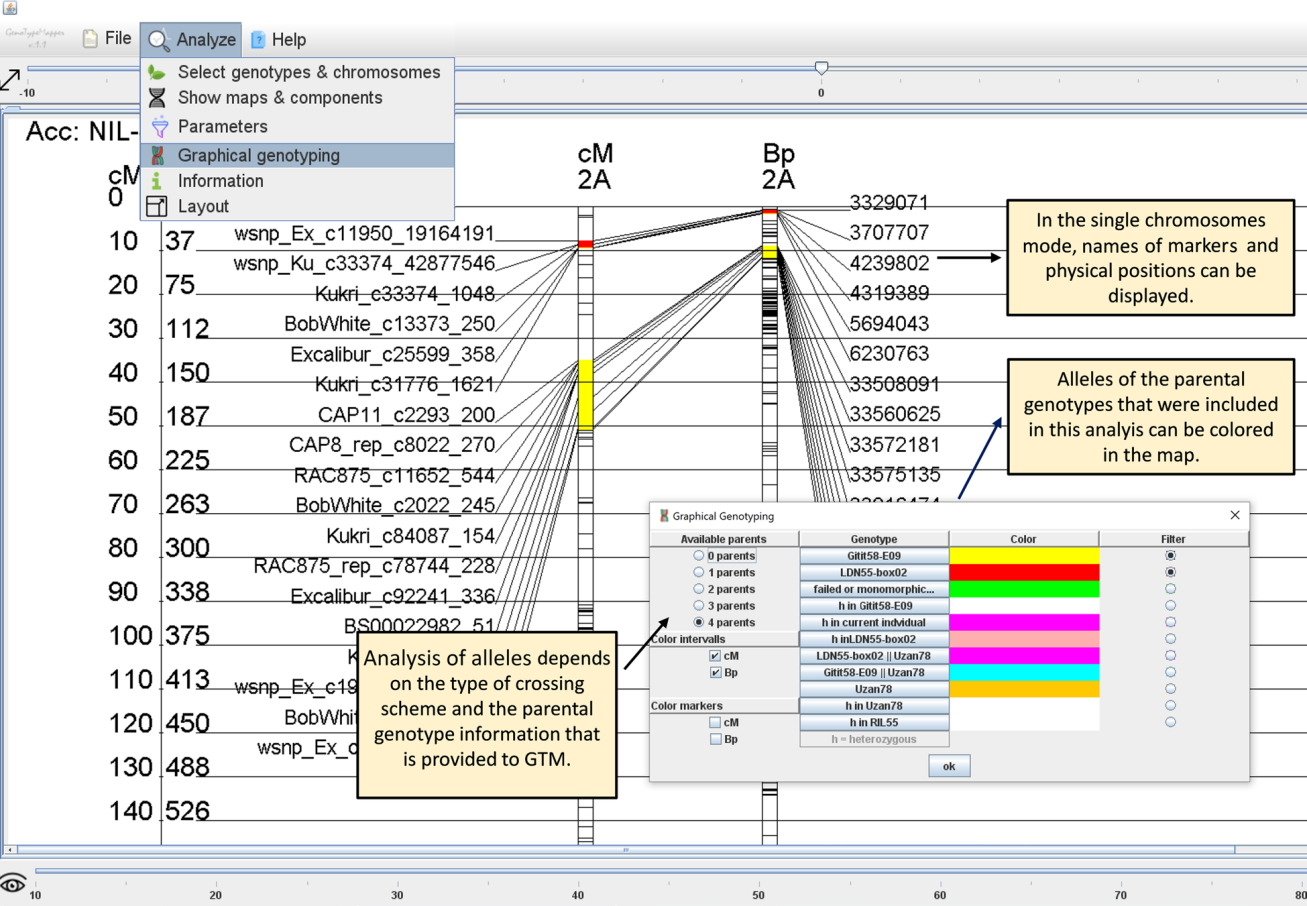
Graphical user interface of GenoTypeMapper in single chromosome mode. In this mode, markers and positions can be displayed. The menu item “Graphical genotyping” allows the user to highlight and analyse markers that were derived from a specific parent. For more detailed information, see the text

After loading input data via the “File” menu, a small dialogue pops up. This dialogue allows the user to select the genotypes and chromosomes of interest. In default mode, a maximum of ten individuals and an arbitrary number of chromosomes can be selected. The alternative single *chromosome mode* is activated by selecting only a single genotype and chromosome. Subsequently, GTM parameters can be adapted in the “Analyse” menu via the menu items “Show maps and components”, “Parameters” and “Graphical genotyping”. The first menu item allows the user to select the information that shall be displayed, such as the genetic- and or physical map. Please note that some additional components, such as marker- and loci names, can only be displayed in the *chromosome mode.* “Parameters” allows the adaptation of the illustrations by modifying variables such as the zooming factor or the spacing between the physical and genetic map. Illustrations can be enlarged up to ten or 30 times in the *default mode* or *chromosome mode*, respectively. In addition to this, the physical size of the same genome can be adapted relative to the genetic map of the chromosome by dividing the physical genome by a user defined factor. Finally, the last menu item, “Graphical Genotyping”, allows the analysis and interpretation of the data that are specified from the sixth to the last column of the input file to determine the origin of alleles in the respective individuals of interest (IOIs). Working scenarios in the respective modes are illustrated in Figs. 2 and 3. Basic information about the number of genotypes that will be subjected to the analysis and the number of available markers and chromosomes are summarized under the menu item “information” (Fig. 2). A small tutorial that guides the user through this process is implemented in the software in the “HELP” menu or available on the website www.genotypemapper.org.

### Allelic discrimination analysis

For each marker, the genotype of the IOI and the information available for parents is compared to determine the origin of its allele. Depending on such a comparison, five, six, ten or eleven different cases might be determined by using genotypic data of either one, two, three or four different parents (Fig. 1). These cases are explored in more detail in Table 2.

**Table 2:** Different allele types that can be determined with GTM relative to the number of parents with genotype information

Parent^a^				IOI^b^	CASE^c^	CASE-description
P1	P2	P3	P4			
A	–	–	–	A	1_1	IOI—allele was derived from parent P1
T	–	–	–	A	1_2	IOI—allele was derived from parent P2
N	–	–	f	N	1_3	Analysis failed because of failed genotypic data
N	–	–	–	H	1_4	Allele in IOI is heterozygous
H	–	–	–	N	1_5	Allele in parent P1 is heterozygous
N	H	–	–	N	2_6	Allele in parent P2 is heterozygous
T	T	A	–	A	3_7	IOI—allele was derived from parent P3
N	N	H	–	N	3_8	Allele in recurrent parent P3 is heterozygous
A	T	T	–	T	3_9	IOI—allele was derived from parent P2 or P3
T	A	T	–	T	3_10	IOI—allele was derived from parent P1 or P3
N	N	N	H	N	4_11	Allele in parent P4 is heterozygous

^a^N = (A, G, T, C), whereas H = (M, R, K, W, S, Y, K) nucleotide ambiguity code.

^b^IOI = individual of interest.

^c^Cases are coded with two numbers and separated by a baseline (x_y), that represents the number of analysed parents (x) and case_types (y). Each case_type is mentioned only once, but case types determined with fewer parents can also be detected with more parents. For more detailed information see the text and Fig. 1.

If polymorphic genotype information from one or two parents of a biparental population is provided, the IOI alleles origin can either be dedicated to parent P1 (case 1_1) or parent P2 (case 1_2) (Fig. 1, Table 2). Genotype information of three or four parents, e.g., of a triple cross, allows the identification of IOI alleles that were derived from the recurrent parent P3 (case 3_7) or from parents P2 /P3 (3_9) or P1/ P3 (3_10) (Fig. 1, Table 2). In addition to this, heterozygosity can be detected in parent P1 (case_ 1_5), parent P2 (case_2_6), parent P3 (case_3_8), parent P4 (case 4_11) (Table 2). Failed or monomorphic data (case 1_3) are filtered out by default to omit mistakes in the allelic discrimination analysis (Table 2).

The colour of each case can be defined by the user to highlight either marker positions and/or marker intervals of adjacent markers that share the same allele information. No colour is added to intervals of adjacent markers with different allele information to omit imprecise representation of the genotypic data (Additional file 2: Figure S2). Markers that belong to a specific allele type of interest can be filtered out and displayed. The image or the markers can be shown on the screen and exported as a PNG image or as a tab delimited text file. The user might change the size and orientation of the image via the menu item “Layout” (Fig. 2).

### Case study—data preparation

All available marker sequences of the 15K iSelect chip [33] were aligned to the wild emmer genome with a local version of the Basic Local Alignment Search Tool (BLAST) v2.10 [34]. Nucleotide BLAST (BLASTn) search was used with the following default parameters:

- Expectation threshold = 10
- Wordsize = 28
- Match/Mismatch score = 1, −2
- Gapcosts = linear
- Filtering of low complexity regions = Yes

Markers with BLASTn-hits on different chromosomes were subsequently compared with the durum consensus map [35] and discarded if they revealed multiple BLASTn hits on the consensus map chromosomes in a distance of more than one 1,000,000 base pairs (bp). A flow chart of this work scheme was added to the attachments (Additional file 3: Figure S3). The 15K iSelect data of the corresponding NILs (NIL-U-2B-1, NIL-B-7A-2) and their recurrent parents (BarNir, Uzan) and crossing parents (F7RIL12, F7RIL55 G18-16 and LDN) were used (TraitGenetics GmbH, Gatersleben, Germany). Genotypic data that did not fail or that were not monomorphic in these eight samples were regarded as putatively informative if the markers could be assigned to a physical or a genetic position (Additional file 3: Figure S3).

## Results and discussion

**Fig. 4:**
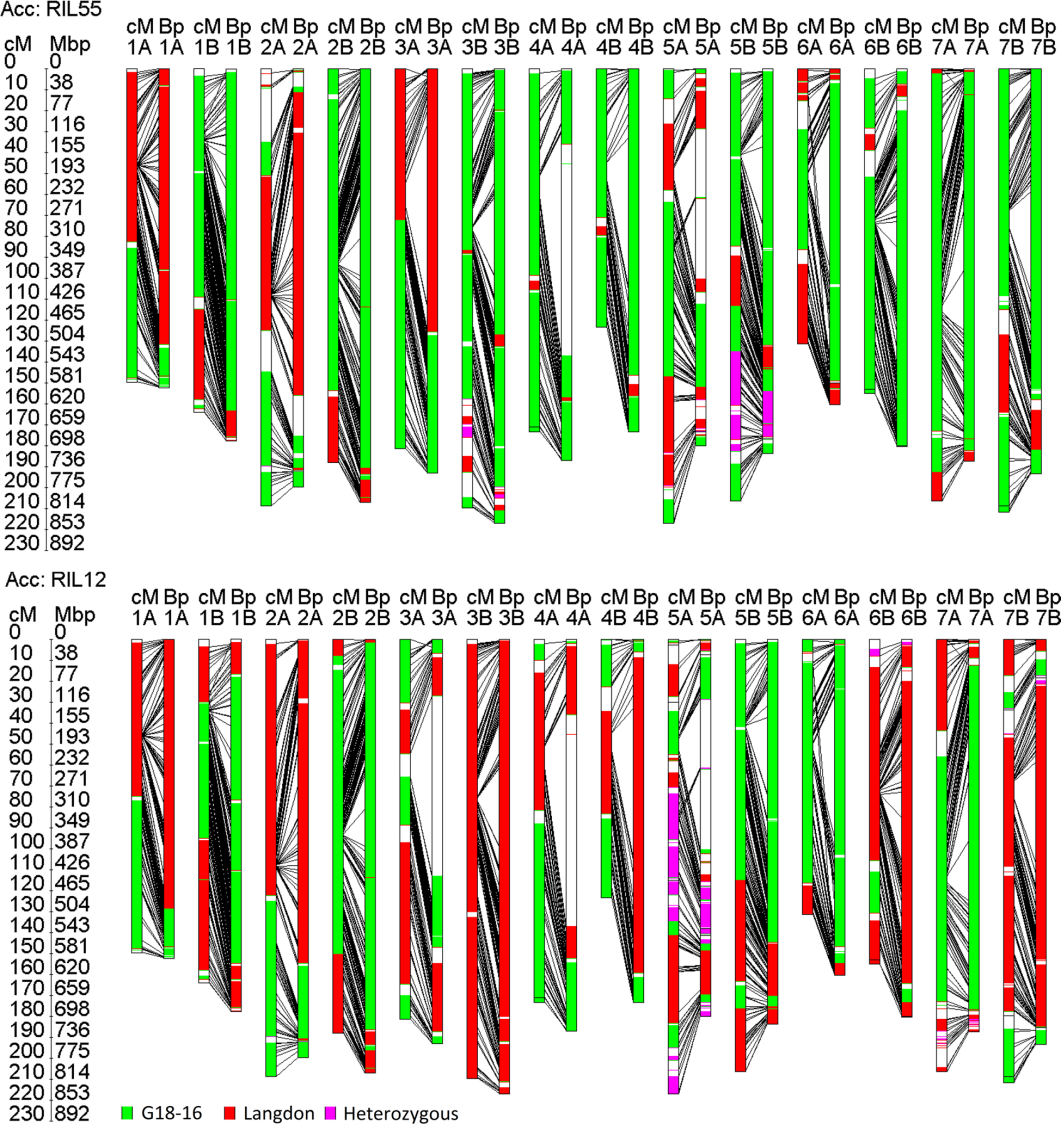
Genetic and physical genotyping of RIL55 and RIL12 with GTM. Both RILs resulted from a biparental cross of the drought-resistant wild emmer accession Gt18-16 and the drought-susceptible durum wheat accession Langdon. Information about the colouring of the respective alleles is illustrated in the figure. For more detailed information, see the text

**Fig. 5:**
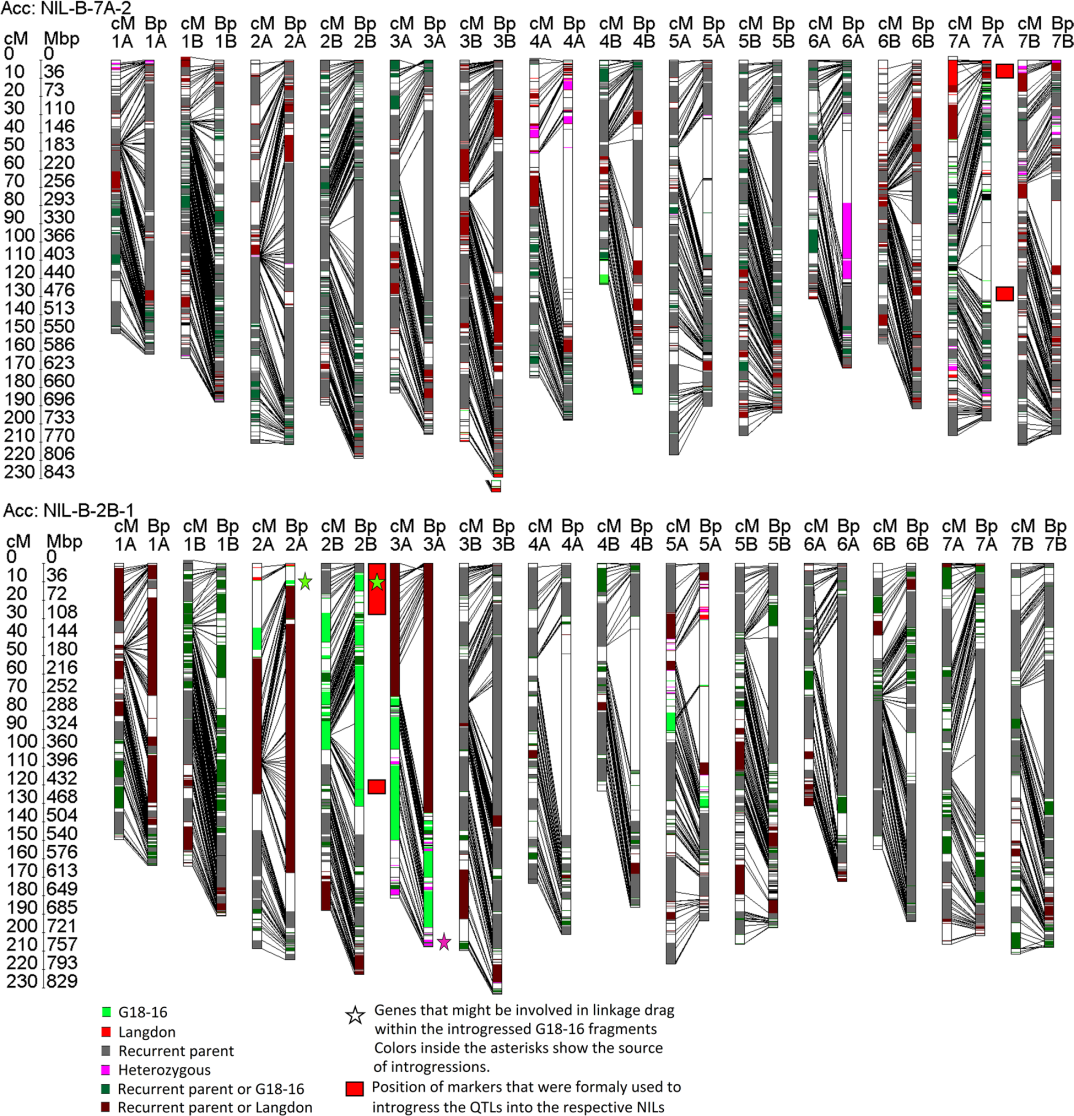
Genetic and physical genotyping of the near isogenic lines NIL-U-2B-1 and NIL-B-7A-2 with GTM. Recurrent parents were the hexaploid bread wheat accession BarNir for NIL-B-7A-2 and the tetraploid durum wheat accession Uzan for NIL-U-2B-1. Information about the colouring of the respective alleles is illustrated in the figure. For more detailed information, see the text

All 12,908 15K iSelect marker sequences [33] were used for BLASTn search (Additional file 3: Figure S3). For 12,445 of these, BLASTn-hits were obtained. A set of 2,813 markers revealed hits to only one chromosome. Eighty-nine markers gave hits to no specific chromosome at all. Out of the remaining 9,632 markers with hits to more than one chromosome, 4,898 markers could be linked to the corresponding genetic chromosomes of the durum consensus map [35]. Finally, sets of 2,495 and 4,898 markers were screened to exclude those with multiple hits on the target chromosomes in a range of more than 1,000,000 bp, leading to a final set of 7,245 markers (Additional file 3: Figure S3, Additional file 4: Table S1 and Additional file 5: Table S2). Two-thousand-one-hundred-twenty of the 7,245 physically anchored iSelect markers were monomorphic or harboured failed genotypic data and were therefore regarded as non-informative. Of the remaining 5,125 markers, 2,971 were polymorphic between G18-16 and LDN. Anchoring the QTL-flanking markers that were previously used to transfer the QTL-regions into the NILs [28] to the physical map of Zavitan [16] revealed that they span over the centromeric region on both chromosomes (Additional file 6: Table S3). In line with these results, about two-third of the wild emmer chromosome 2B and 7A were transferred from F7RIL55 and F7RIL12 (Fig. 4) into NIL-U-2B-1 and NIL-B-7A-2, respectively (Fig. 5). Wild emmer fragments were also detected on chromosome 2A, 3A and 5A in NIL-U-2B-1 and 4B in NIL-B-7A-2 (Fig. 5). Although a remarkable reduction in the genetic background of the wild emmer parent in both NILs was observed at BC3F5 after the MAS procedure (Additional file 1: Figure S1, Fig. 5), these regions might cause linkage drag. To pinpoint regions that are potentially involved in linkage drag, primer pairs of well-described domestication genes [36] that are located on the NIL-U-2B-1 chromosome arms with introgressions from the wild emmer parent (Fig. 5) were aligned to the reference genome of wild emmer. It turned out that NIL-U-2B-1 contains wild emmer alleles of the domestication genes *PpdA1* (TRIDC2AG009040) [37] and *PPdB1* (TRIDC2BG010800) [38] on chromosomes 2AS and 2BS, respectively. In addition to this, a heterozygous version of the so called *Lux* (TRIDC3AG074490) gene, which was described to additively interact with *PPdB1* [39], was identified at the very distal end of chromosome 3AL. Notably, NIL-U-2B-1 was also shown to suffer from delayed heading time [28]. Presence/absence analysis of the respective regions in future segmental RILs might allow the identification of the exact causative loci. The allelic origin of other popular domestication genes, such as *VrnA1* [38] or Q on chromosome 5A [40] or the popular dwarfing gene *RhtB1* [41], were also tested. However, none of these was transferred from G18-16 into the NILs.

## Conclusions

This paper describes the development of software, that easily allows the display and comparison of genotypic data on genetic and physical maps by including a maximum amount of marker information, which is usually removed at the very early stages of the mapping procedure.

We presented a case study elucidating the use of GTM by using genotypic information of NILs carrying introgressions of wild emmer wheat, aiming to improve drought resistance in wheat cultivars [28, 29]. Both NILs were shown to be more tolerant to drought than their recurrent parents but showed phenotypes that may stem from a linkage drag, e.g., an increased plant height or an increased number of days to heading (DPH) [28, 29]. GTM enabled the visualization of the genotypic and physical results of a MAS procedure, which started from wild emmer wheat into tetra- or hexaploid wheat cultivars (Additional file 1: Figure S1). It depicts the special relevance and usability of physical and genetic genotyping for pre-breeding purposes to handle linkage drag and heterozygosity transferred from crop wild relatives.

Marker information illustrated by GTM is the product of the displayed genotypes, the chromosomes and the number of markers analysed. In the current version of the program, a maximum of ten genotypes can be displayed on the screen in the *chromosome mode*. If more than one genotype is analysed, the allelic discrimination analysis needs to be repeated for each individual. GTM uses multithreading to handle these situations efficiently.

To doublecheck GTM computational performance, artificial datasets with 3083, 6166, 9249 and 12,332 markers were created and used to explore GTM performance.

On a computer with an Intel five processor of the 8^th^ generation, GTM can analyse a single genotype with 3,083 polymorphic markers on 14 chromosomes in less than 752 ms (ms) (Additional file 7: Figure S4). Ten individuals with the same amount of markers can be analysed in 1,429 ms, which is approximately twice as much of the time that is needed for a single individual (Additional file 7: Figure S4). The computation time increases linearly with the amount of analysed individuals. In addition to this, the number of markers has a significant impact on GTM computation time per individual and might hamper its performance if more than a single individual is analysed in parallel (Additional file 7: Figure S4). The tests were run under the standard conditions of the 1.8.0_231 JRE with a maximum heap space of 268,435,456 Byte.

We conclude that GTM can easily analyse up to ten genotypes on datasets with up to 6,000–7,000 markers in less than six seconds (Additional file 7: Figure S4). Analysing more markers with GTM is possible but requires more computation time. In such a case, we recommend analysing fewer genotypes and/or chromosomes in parallel.

Currently, genotypic data of 500 individuals with up to 50,000 markers can be uploaded to the software.

GTM’s allelic discrimination analysis works accurate, but sometimes different allele information might appear at the same genetic position, e.g., if consensus marker positions are used. In those cases, adequate and reliable predictions about the correct corresponding allele type are theoretically not possible/difficult and require the user’s expertise (Additional file 2: Figure S2).

We illustrate how such an analysis might be performed in detail (Additional file 3: Figure S3) with two and four different parents (Figs. 4 and 5). One additional example that illustrates how genotyping analysis might be performed if genotype information of only a single parent is available (Fig. 1 and Table 2) is given in the attachment for a NIL that carries an introgression harbouring the leaf rust resistance gene *Lr1* [42] (Additional file 8: Figure S5). Markers in this example were anchored to genetic positions via the wheat consensus map [43]. Positions of the markers in the Chinese Spring genome are publicly available [44, 45].

In the case of this study, only 7,245, i.e., merely 60% of the 12,908 iSelect markers, could be assigned to reliable physical positions. This is very likely due to the following reasons: (i) part of the iSelect markers are designed based on D genome sequences; (ii) markers might have been assigned to different chromosomes in the Zavitan genome than in the durum consensus map; (iii) iSelect markers deliver bad BLAST-hits that likely fall below filtering criteria, and (iv) markers of gene families residing on several chromosomes and/or on homologous chromosomes on the AA and BB genomes cannot be assigned to a clear physical position.

Please note that for many BeadArrays or Gene chips, physical positions on the reference genomes are provided on the Illumina website (www.illumina.com) (e.g., maize) or Affymetrix website (www.Affymterix.com) (e.g., strawberry, apple, wheat, cotton, soybean, maize). In addition to this, physical positions of diverse marker types can also be obtained from databases, such as GrainGenes [45]. In such cases, the identification of physical positions of the markers is not required. Anchoring of markers to physical position in the genome can also easily be accomplished with a simple BLASTN- search. In some cases, this approach might be sufficient [46].

GTM also offers the possibility to analyse single genetic or physical maps. In addition to this, two genetic maps can be compared. Respective test datasets are available on www.genotypemapper.org. Please note that GTM can also be used for other organisms than plants. As an example, we genotyped two F2 pig individuals that were derived from a cross between a “Göttingen Minipig” boar and “Yorkshire” gilt [47]. The results were added to the supplement of this article (Additional file 9: Figure S6). We conclude that the software can be used for human and animal genetics as well.

## Availability and requirements

Project name: GenoTypeMapper (GTM).

Project home: www.genotypemapper.org

Operating system(s): Platform independent

Programming language: Java

Other requirements: Java Runtime Environment 1.8.0_231 (or higher)

License: End use license agreements (EULA)

Any restrictions to use by non-academics: no

## Supplementary information

**Additional file 1: Figure S1.** Establishment of near isogenic lines carrying wild emmer QTLs for drought stress tolerance. (1) The wild emmer acc. G18-16 was crossed with the cv. Langdon (LDN) to obtain a segregating F6 mapping population. A map with 197 microsatellites and 493 DArT markers on 690 loci was constructed to identify QTLs for drought stress tolerance associated traits [30, 31]. (2/3) Markers that flank the QTL-regions of interest were then used to introgress the QTLs into the background of elite wheat cultivars. The plants were subsequently backcrossed three times to diminish the genomic fraction of the donor parent. Finally, BC3F3 and BC3F4 progenies were phenotyped under water-limited and well-watered conditions together with their recurrent parents [28]. NIL-U-2B-1 and NIL-B-7A-2 not only showed better tolerance than their recurrent elite parents but also exhibited negative traits [28]. (4) 15k iSelect genotypic data of F7 descendants of the original mapping population are used to reconstruct a new high-resolution genetic map [32]. Green and purple boxes represent male and female plants, respectively.

**Additional file 2: Figure S2.** Interval- and marker-based colouring in GTM. (A) In a classical genetic mapping procedure, heterozygous & monomorphic markers of parents are discarded. The remaining molecular markers form linkage groups of the same allele types so that GTM can highlight intervals that are flanked by markers with the same allele information. Intervals that are flanked by markers with different allele types are not coloured because the exact position of recombination between both markers is unknown. (B) If genetic consensus marker positions are used, markers with different allele information can be assigned to the same genetic position. In those cases, colouring of intervals is not possible, and marker-based colouring might lead to inaccurate results (see Marker M1, M2, M3). Filtering of the allele types of interest might be the method of choice to analyse that kind of genotype, as illustrated in (C) and (D).

**Additional file 3: Figure S3.** BLAST-scheme that illustrates the assignment of physical loci of 15k-iSelect markers in the Triticum turgidum ssp. dicoccoides genome. Numbers obtained in the analysis are coloured in orange. For more details, see text.

**Additional file 4: Table S1.** 15k iSelect -markers that were assigned to physical or genetic loci.

**Additional file 5: Table S2.** In total, 7,245 iSelect markers with physical and genetic positions.

**Additional file 6: Table S3.** Genetic and physical positions of flanking microsatellite and DArT markers of QTL regions 2BS and 7AS.

**Additional file 7: Figure S4.** GTM’s performance relative to the number of markers and genotypes that are analysed.

**Additional file 8: Figure S5.** Allelic discrimination analysis with one parent in GenoTypeMapper. Wheat near isogenic line Tc-Lr1 with an introgression of the leaf rust resistance gene Lr1 in the background of the parent “Thatcher” on chromosome 5DL. Only genotype data of the Thatcher line was available, so allele analysis with one parent was performed (Figure 2, Table 2). Please notice that monomorphic markers between Thatcher and the second parent were not detected due to the missing genotype information of one parent. Therefore, filtering and colouring of the introgressed markers was the method of choice to illustrate the introgressed regions. This analysis illustrates that introgressions of the unknown parent also occurred to other chromosomes. The physical genome shows that centromeric regions were introgressed from the resistant parent into the NIL. Please note: This test-dataset is quite large and comprises more than 30540 markers.

**Additional file 9: Figure S6.** Physical genotyping of two F2 pigs: The F2 boars resulted from a cross of a Minipig (MP) (boar) and a Yorkshire (YS) (gilt). In the parental generation seven purebred Yorkshire (YY) sows were mated to 14 Goettingen Minipig (MM) boars. Among the YM F1 animals, 26 gilts and 13 boars were mated to produce 279 F2 animals. Genotypic data (60k iSelect data) for the animals were previously published and reused in this analysis [47]. The marker data for this illustration were obtained in two steps: First, consensus marker information from the parental YS boars and MP gilts was extracted. Then, markers that failed in YS or MP were removed. Information about the colouring of respective alleles is illustrated in the figure. It should be pointed out, that the Y chromosome is not shown due to the fact that it has to be derived from the MP.

## Data Availability

The program, user manual and example data sets are freely available on https://www.genotypemapper.org.

## Abbreviations

BLAST: Basic locale alignment search tool
Bp: Basepairs
cM: Centimorgan
DPH: Days planting to heading
GTM: GenoTypeMapper
LDN: Langdon (name of the drought susceptible durum cultivar)
LOD: Logarithm of odds
MAS: Marker assisted selection
NIL: Near isogenic line
UI: User interface
