# Supplementary material for: GenoTypeMapper: graphical genotyping on genetic and sequence-based maps
Source: Plant Methods. 2020 Sep 10;16:123. doi: 10.1186/s13007-020-00665-7 (PMC7488165; doi:10.1186/s13007-020-00665-7)
Supplement: Supplementary file 1 — Additional file 1: Figure S1. Establishment of near isogenic lines carrying wild emmer QTLs for drought stress tolerance. (1) The wild emmer acc. G18-16 was crossed with the cv. Langdon (LDN) to obtain a segregating F6 mapping population. A map with 197 microsatellites and 493 DArT markers on 690 loci was constructed to identify QTLs for drought stress tolerance associated traits [30, 31]. (2/3) Markers that flank the QTL-regions of interest were then used to introgress the QTLs into the background of elite wheat cultivars. The plants were subsequently backcrossed three times to diminish the genomic fraction of the donor parent. Finally, BC3F3 and BC3F4 progenies were phenotyped under water-limited and well-watered conditions together with their recurrent parents [28]. NIL-U-2B-1 and NIL-B-7A-2 not only showed better tolerance than their recurrent elite parents but also exhibited negative traits [28]. (4) 15k iSelect genotypic data of F7 descendants of the original mapping population are used to reconstruct a new high-resolution genetic map [32]. Green and purple boxes represent male and female plants, respectively. [file 13007_2020_665_MOESM1_ESM.docx]

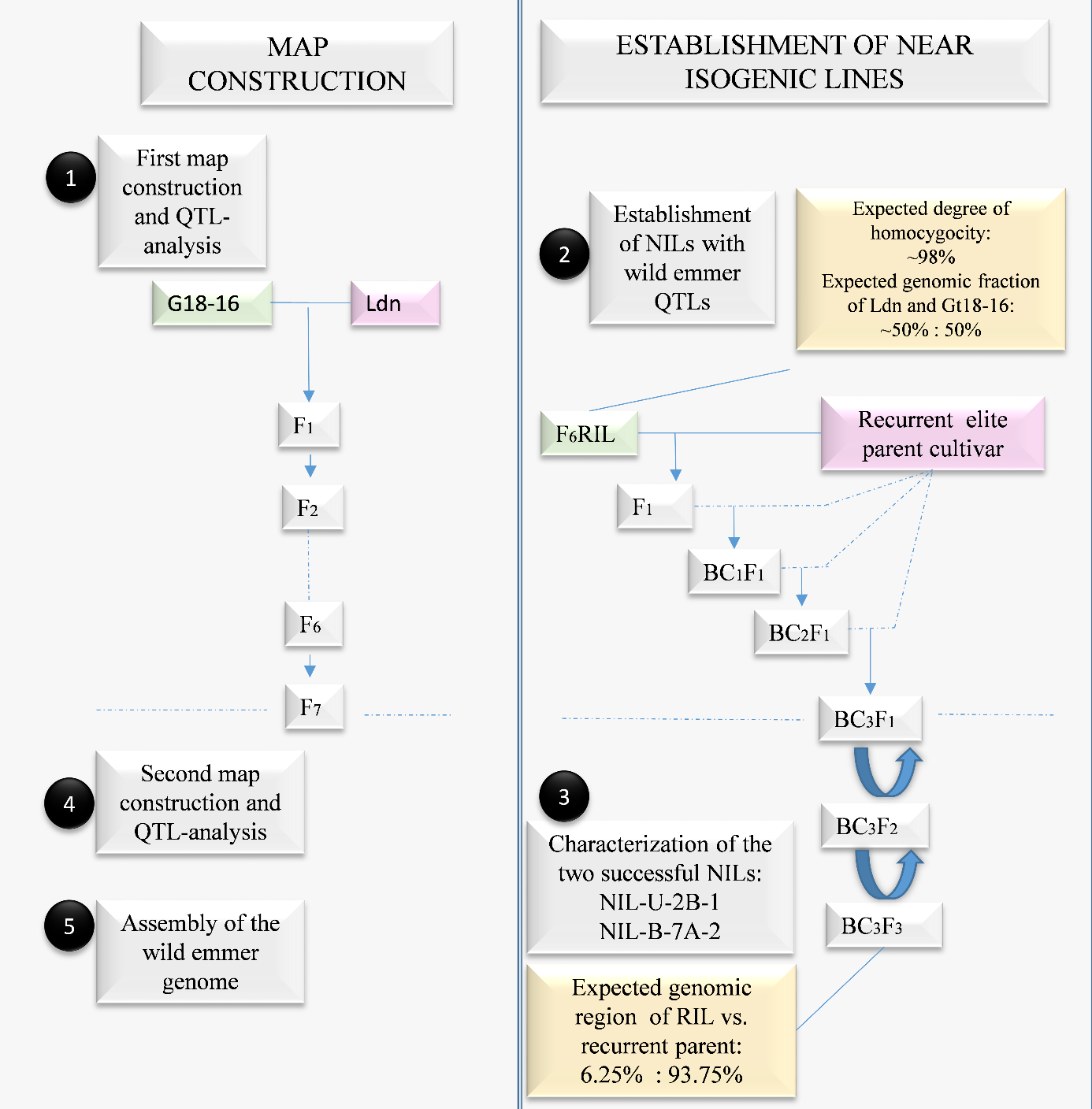


**Figure S1: Establishment of near isogenic lines carrying wild emmer QTLs for drought stress tolerance**. (1) The wild emmer acc. G18-16 was crossed with the cv. Langdon (LDN) to obtain a segregating F6 mapping population. A map with 197 microsatellites and 493 DArT markers on 690 loci was constructed to identify QTLs for drought stress tolerance associated traits [30, 31]. (2/3) Markers that flank the QTL-regions of interest were then used to introgress the QTLs into the background of elite wheat cultivars. The plants were subsequently backcrossed three times to diminish the genomic fraction of the donor parent. Finally, BC3F3 and BC3F4 progenies were phenotyped under water-limited and well-watered conditions together with their recurrent parents [28]. NIL-U-2B-1 and NIL-B-7A-2 not only showed better tolerance than their recurrent elite parents but also exhibited negative traits [28]. (4) 15k iSelect genotypic data of F7 descendants of the original mapping population are used to reconstruct a new high-resolution genetic map [32]. Green and purple boxes represent male and female plants, respectively.
