# Supplementary material for: GenoTypeMapper: graphical genotyping on genetic and sequence-based maps
Source: Plant Methods. 2020 Sep 10;16:123. doi: 10.1186/s13007-020-00665-7 (PMC7488165; doi:10.1186/s13007-020-00665-7)
Supplement: Supplementary file 2 — Additional file 2: Figure S2. Interval- and marker-based colouring in GTM. (A) In a classical genetic mapping procedure, heterozygous & monomorphic markers of parents are discarded. The remaining molecular markers form linkage groups of the same allele types so that GTM can highlight intervals that are flanked by markers with the same allele information. Intervals that are flanked by markers with different allele types are not coloured because the exact position of recombination between both markers is unknown. (B) If genetic consensus marker positions are used, markers with different allele information can be assigned to the same genetic position. In those cases, colouring of intervals is not possible, and marker-based colouring might lead to inaccurate results (see Marker M1, M2, M3). Filtering of the allele types of interest might be the method of choice to analyse that kind of genotype, as illustrated in (C) and (D). [file 13007_2020_665_MOESM2_ESM.docx]

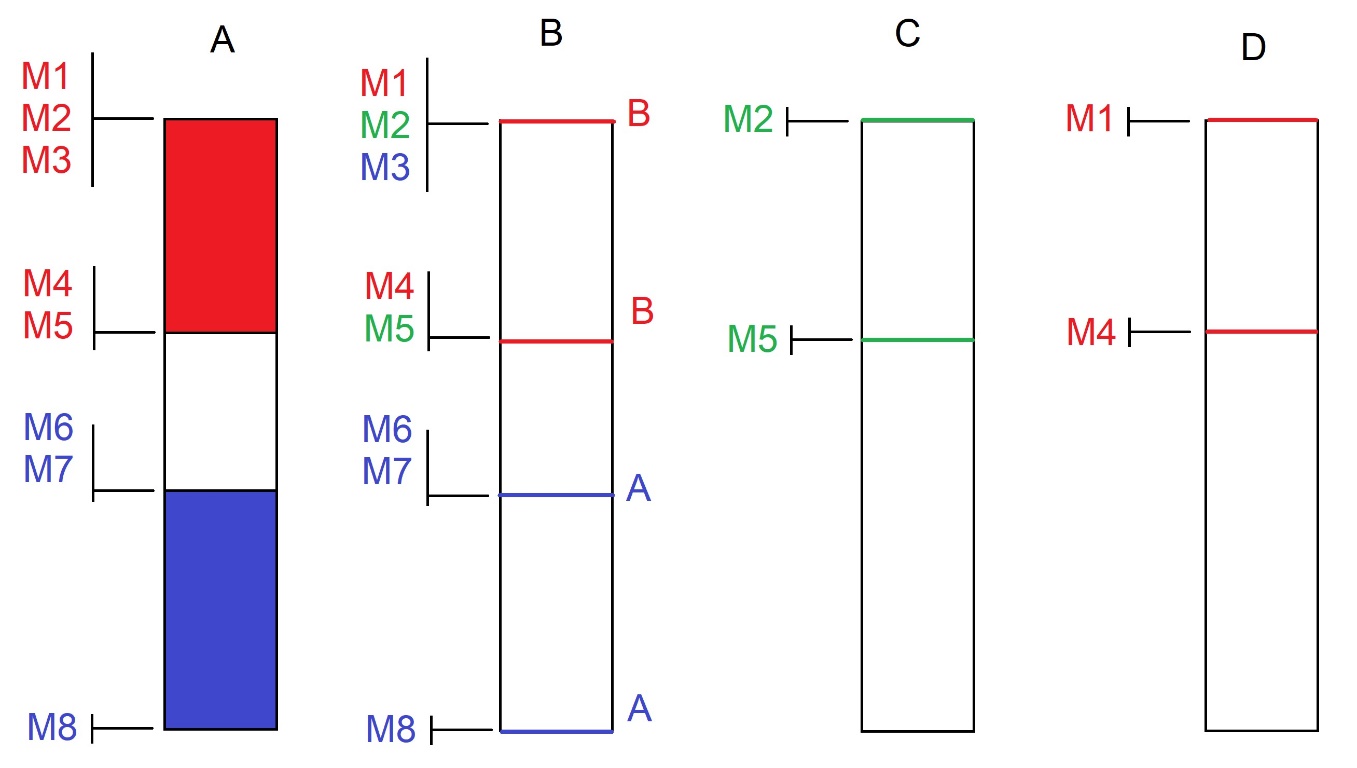


**Figure S2: Interval- and marker-based colouring in GTM.** (A) In a classical genetic mapping procedure, heterozygous & monomorphic markers of parents are discarded. The remaining molecular markers form linkage groups of the same allele types so that GTM can highlight intervals that are flanked by markers with the same allele information. Intervals that are flanked by markers with different allele types are not coloured because the exact position of recombination between both markers is unknown. (B) If genetic consensus marker positions are used, markers with different allele information can be assigned to the same genetic position. In those cases, colouring of intervals is not possible, and marker-based colouring might lead to inaccurate results (see Marker M1, M2, M3). Filtering of the allele types of interest might be the method of choice to analyse that kind of genotype, as illustrated in (C) and (D).
