# Supplementary material for: GenoTypeMapper: graphical genotyping on genetic and sequence-based maps
Source: Plant Methods. 2020 Sep 10;16:123. doi: 10.1186/s13007-020-00665-7 (PMC7488165; doi:10.1186/s13007-020-00665-7)
Supplement: Supplementary file 3 — Additional file 3: Figure S3. BLAST-scheme that illustrates the assignment of physical loci of 15k-iSelect markers in the Triticum turgidum ssp. dicoccoides genome. Numbers obtained in the analysis are coloured in orange. For more details, see text. [file 13007_2020_665_MOESM3_ESM.docx]

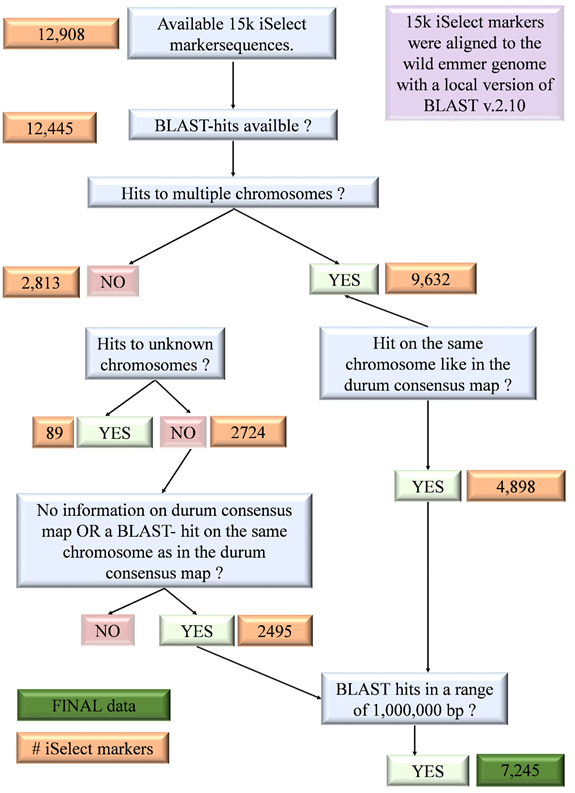


**Figure S3: BLAST-scheme that illustrates the assignment of physical loci of 15k-iSelect markers in the *Triticum turgidum* ssp. *dicoccoides* genome***.* Numbers obtained in the analysis are coloured in orange. For more details, see text.
