# Supplementary material for: GenoTypeMapper: graphical genotyping on genetic and sequence-based maps
Source: Plant Methods. 2020 Sep 10;16:123. doi: 10.1186/s13007-020-00665-7 (PMC7488165; doi:10.1186/s13007-020-00665-7)
Supplement: Supplementary file 4 — Additional file 4: Table S1. 15k iSelect -markers that were assigned to physical or genetic loci. [file 13007_2020_665_MOESM4_ESM.docx]

Table S1: 15k iSelect -markers that were assigned to physical or genetic loci.

| **Assigned to:** | **Physical***  **# positions & markers**  **# markers** | **Genetic **** | |
| --- | --- | --- | --- |
| **Chr.** |  | **# markers** | **# positions** |
| 1A | 421 | 350 | 157 |
| 2A | 465 | 384 | 134 |
| 3A | 391 | 339 | 131 |
| 4A | 305 | 259 | 124 |
| 5A | 504 | 445 | 174 |
| 6A | 472 | 403 | 125 |
| 7A | 564 | 492 | 192 |
| 1B | 668 | 601 | 207 |
| 2B | 795 | 720 | 229 |
| 3B | 618 | 545 | 206 |
| 4B | 269 | 241 | 115 |
| 5B | 646 | 562 | 212 |
| 6B | 603 | 532 | 174 |
| 7B | 524 | 480 | 175 |
| **Sum** | **7245** | **6353** | **2355** |

* Physical positions were obtained from the wild emmer genome Zavitan [16]

** Genetic positions were obtained from the durum wheat consensus map [35]. For more details, see text and Additional file 3.
