# Supplementary material for: GenoTypeMapper: graphical genotyping on genetic and sequence-based maps
Source: Plant Methods. 2020 Sep 10;16:123. doi: 10.1186/s13007-020-00665-7 (PMC7488165; doi:10.1186/s13007-020-00665-7)
Supplement: Supplementary file 6 — Additional file 6: Table S3. Genetic and physical positions of flanking microsatellite and DArT markers of QTL regions 2BS and 7AS. [file 13007_2020_665_MOESM6_ESM.docx]

**Table S3: Genetic and physical positions of flanking microsatellite and DArT markers of QTL regions 2BS and 7AS.**

| QTL | Marker* | previous genetic map | Primer | Genetic positions **  (cM) | Physical position ***  (bp) | |
| --- | --- | --- | --- | --- | --- | --- |
| 2BS | **Xgwm1128(N)** | 24.8 | NA | NA | NA | NA |
|  |  |  |  |  | NA | NA |
|  | **Xwmc35 (M)** | 64.6 |  | NA | 119525354 | 119525241 |
|  | **Xgwm1177(S)** | 78.9 | NA | RAC875_c9122_395 (Up)  Kukri_c110148_53 (down) | 431218016 |  |
|  |  |  |  |  | 431216039 | 431216139 |
| Interval | | 54,1 |  | | >431216139 | |
| 7AS | **gwm60(N)** | 0 | AV | NA | 47659947 | 47659929 |
|  |  |  |  | NA | 47659732 | 47659750 |
|  | **Xwmc596(S)** | 48 | AV | NA | 473621980 | 473621961 |
|  |  |  |  | NA | 473621591 | 473621611 |
| Interval | | 48.0 |  | | 425961664 | |

*) Markers located at the northern (N), southern (S) and in the middle (M) of the QTL-interval were used for the introgression of the corresponding QTL-fragments into the near isogenic lines [28]. (NA = Not available, AV = available).

**) Genetic positions were obtained from the durum wheat consensus map [35].

***) Physical positions were obtained from the wild emmer genome cv. Zavitan [16]
