# Supplementary material for: GenoTypeMapper: graphical genotyping on genetic and sequence-based maps
Source: Plant Methods. 2020 Sep 10;16:123. doi: 10.1186/s13007-020-00665-7 (PMC7488165; doi:10.1186/s13007-020-00665-7)
Supplement: Supplementary file 7 — Additional file 7: Figure S4. GTM’s performance relative to the number of markers and genotypes that are analysed. [file 13007_2020_665_MOESM7_ESM.docx]

**Figure S4: GTM’s performance relative to the number of markers and genotypes that are analysed.**
