# Supplementary material for: GenoTypeMapper: graphical genotyping on genetic and sequence-based maps
Source: Plant Methods. 2020 Sep 10;16:123. doi: 10.1186/s13007-020-00665-7 (PMC7488165; doi:10.1186/s13007-020-00665-7)
Supplement: Supplementary file 8 — Additional file 8: Figure S5. Allelic discrimination analysis with one parent in GenoTypeMapper. Wheat near isogenic line Tc-Lr1 with an introgression of the leaf rust resistance gene Lr1 in the background of the parent “Thatcher” on chromosome 5DL. Only genotype data of the Thatcher line was available, so allele analysis with one parent was performed (Figure 2, Table 2). Please notice that monomorphic markers between Thatcher and the second parent were not detected due to the missing genotype information of one parent. Therefore, filtering and colouring of the introgressed markers was the method of choice to illustrate the introgressed regions. This analysis illustrates that introgressions of the unknown parent also occurred to other chromosomes. The physical genome shows that centromeric regions were introgressed from the resistant parent into the NIL. Please note: This test-dataset is quite large and comprises more than 30540 markers. [file 13007_2020_665_MOESM8_ESM.docx]

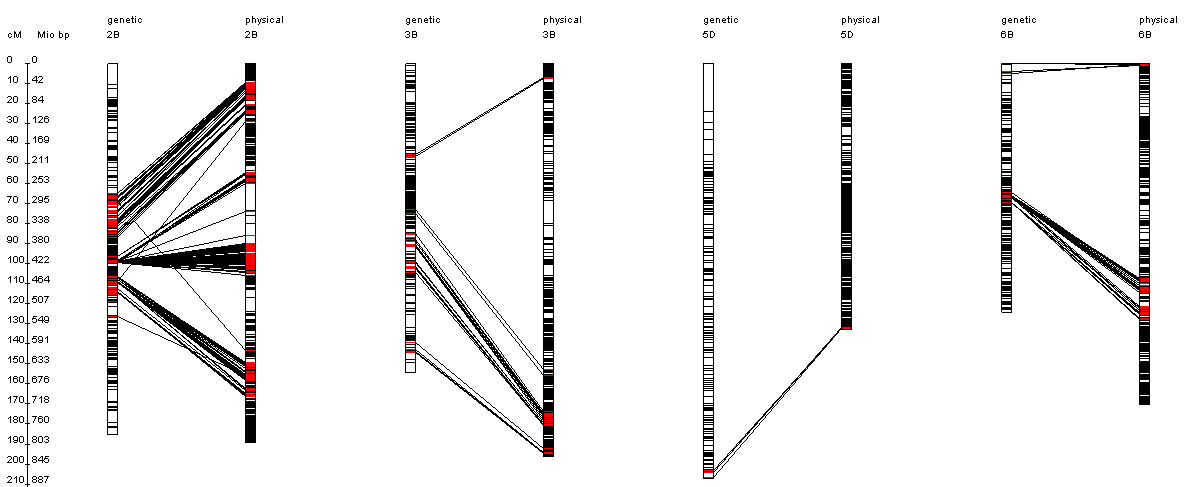


**Figure S5: Allelic discrimination analysis with one parent in GenoTypeMapper**. Wheat near isogenic line Tc-Lr1 with an introgression of the leaf rust resistance gene *Lr1* in the background of the parent “Thatcher” on chromosome 5DL. Only genotype data of the Thatcher line was available, so allele analysis with one parent was performed (Figure 2, Table 2). Please notice that monomorphic markers between Thatcher and the second parent were not detected due to the missing genotype information of one parent. Therefore, filtering and colouring of the introgressed markers was the method of choice to illustrate the introgressed regions. This analysis illustrates that introgressions of the unknown parent also occurred to other chromosomes. The physical genome shows that centromeric regions were introgressed from the resistant parent into the NIL. *Please note: This test-dataset is quite large and comprises more than 30540 markers.*
