# Supplementary material for: GenoTypeMapper: graphical genotyping on genetic and sequence-based maps
Source: Plant Methods. 2020 Sep 10;16:123. doi: 10.1186/s13007-020-00665-7 (PMC7488165; doi:10.1186/s13007-020-00665-7)
Supplement: Supplementary file 9 — Additional file 9: Figure S6. Physical genotyping of two F2 pigs: The F2 boars resulted from a cross of a Minipig (MP) (boar) and a Yorkshire (YS) (gilt). In the parental generation seven purebred Yorkshire (YY) sows were mated to 14 Goettingen Minipig (MM) boars. Among the YM F1 animals, 26 gilts and 13 boars were mated to produce 279 F2 animals. Genotypic data (60k iSelect data) for the animals were previously published and reused in this analysis [47]. The marker data for this illustration were obtained in two steps: First, consensus marker information from the parental YS boars and MP gilts was extracted. Then, markers that failed in YS or MP were removed. Information about the colouring of respective alleles is illustrated in the figure. It should be pointed out, that the Y chromosome is not shown due to the fact that it has to be derived from the MP. [file 13007_2020_665_MOESM9_ESM.docx]

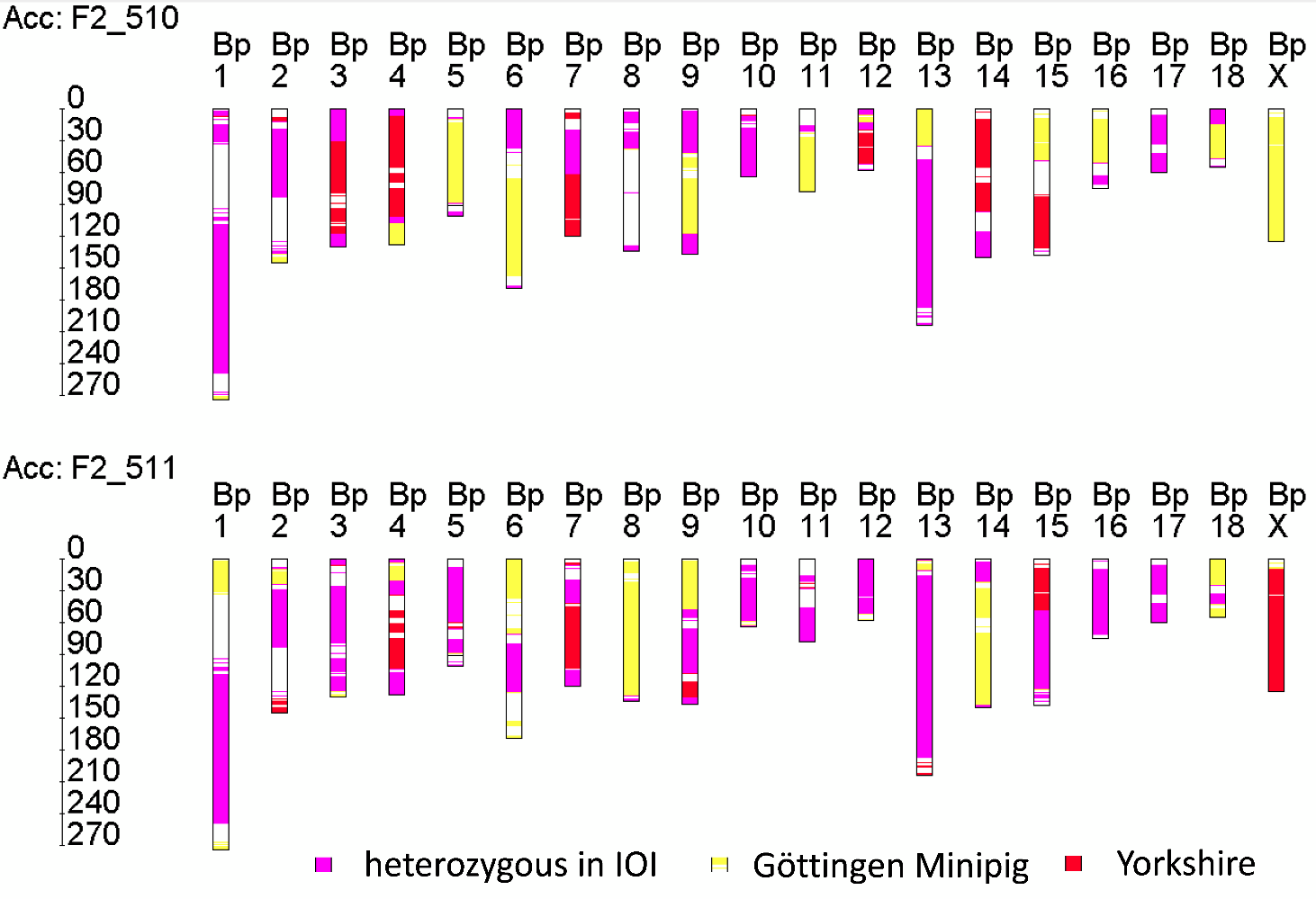


**Figure S6: Physical genotyping of two F2 pigs**: The F**_2_** boars resulted from a cross of a Minipig (MP) (boar) and a Yorkshire (YS) (gilt). In the parental generation seven purebred Yorkshire (YY) sows were mated to 14 Goettingen Minipig (MM) boars. Among the YM F_1_ animals, 26 gilts and 13 boars were mated to produce 279 F_2_ animals. Genotypic data (60k iSelect data) for the animals were previously published and reused in this analysis [47]. The marker data for this illustration were obtained in two steps: First, consensus marker information from the parental YS boars and MP gilts was extracted. Then, markers that failed in YS or MP were removed. Information about the colouring of the respective alleles is illustrated in the figure. It should be pointed out, that the Y chromosome is not shown because it must be derived from the MP.
